# Reduced slow-wave activity and autonomic dysfunction during sleep precede cognitive deficits in Alzheimer’s disease transgenic mice

**DOI:** 10.1038/s41598-023-38214-6

**Published:** 2023-07-11

**Authors:** Chieh-Wen Chen, Yam-Ting Kwok, Yu-Ting Cheng, Yu-Shan Huang, Terry B. J. Kuo, Cheng-Han Wu, Pei-Jing Du, Albert C. Yang, Cheryl C. H. Yang

**Affiliations:** 1 Institute of Brain Science, Brain Research Center, and Sleep Research Center, National Yang Ming Chiao Tung University, No. 155, Sec. 2, Li-Nong St., Taipei, 11221 Taiwan; 2 Sleep Research Center, National Yang Ming Chiao Tung University, Taipei, Taiwan; 3 Brain Research Center, National Yang Ming Chiao Tung University, Taipei, Taiwan; 4 Institute of Brain Science, Digital Medicine and Smart Healthcare Research Center, National Yang Ming Chiao Tung University, No. 155, Sec. 2, Li-Nong St., Taipei, 11221 Taiwan; 5 Department of Neurology, Far Eastern Memorial Hospital, New Taipei, Taiwan; 6 Department of Education and Research, Taipei City Hospital, Taipei, Taiwan; 7 Department of Anesthesiology, Taipei Veterans General Hospital, Taipei, Taiwan; 8 Department of Medical Research, Taipei Veterans General Hospital, Taipei, Taiwan; 9 Center for Mind and Brain Medicine, Tsaotun Psychiatric Center, Ministry of Health and Welfare, Nantou, Taiwan; 10 Department of Health and Leisure Management, Yuanpei University of Medical Technology, Hsinchu, Taiwan

**Keywords:** Heart failure, Translational research

## Abstract

Occurrence of amyloid-β (Aβ) aggregation in brain begins before the clinical onset of Alzheimer’s disease (AD), as preclinical AD. Studies have reported that sleep problems and autonomic dysfunction associate closely with AD. However, whether they, especially the interaction between sleep and autonomic function, play critical roles in preclinical AD are unclear. Therefore, we investigated how sleep patterns and autonomic regulation at different sleep–wake stages changed and whether they were related to cognitive performance in pathogenesis of AD mice. Polysomnographic recordings in freely-moving APP/PS1 and wild-type (WT) littermates were collected to study sleep patterns and autonomic function at 4 (early disease stage) and 8 months of age (advanced disease stage), cognitive tasks including novel object recognition and Morris water maze were performed, and Aβ levels in brain were measured. APP/PS1 mice at early stage of AD pathology with Aβ aggregation but without significant differences in cognitive performance had frequent sleep–wake transitions, lower sleep-related delta power percentage, lower overall autonomic activity, and lower parasympathetic activity mainly during sleep compared with WT mice. The same phenomenon was observed in advanced-stage APP/PS1 mice with significant cognitive deficits. In mice at both disease stages, sleep-related delta power percentage correlated positively with memory performance. At early stage, memory performance correlated positively with sympathetic activity during wakefulness; at advanced stage, memory performance correlated positively with parasympathetic activity during both wakefulness and sleep. In conclusion, sleep quality and distinction between wake- and sleep-related autonomic function may be biomarkers for early AD detection.

## Introduction

Alzheimer’s disease (AD) is the most common cause of dementia and is characterized by progressive memory loss, especially that of declarative memory. More than 6 million people in the United States are affected by AD; unfortunately, its underlying etiology is not yet fully understood^1^. The neuropathological hallmarks of AD are characterized by the presence of extracellular amyloid-β (Aβ) depositions and neurofibrillary tangles. According to numerous previous studies, it has been suggested that the pathogenesis of AD may initiate with the abnormal processing of amyloid precursor protein (APP), which then leads to excessive production or decreased clearance of Aβ in the cortex^2^. The generation of Aβ aggregation in the human brain may begin a decade or more before the clinical symptoms of AD appear; at this time, a cascade of abnormal tau aggregation, neuroinflammation, synaptic dysfunction, cell death, and brain shrinkage occurs^3,4^. For humans with Aβ aggregation in the brain who are clinically asymptomatic (preclinical AD), studies have reported reduced functional connectivity in brain networks affected by amyloid deposition^5,6^, suggesting that other functional changes may occur during the preclinical phase of AD^7^. An understanding of the clinical features that can be measured by convenient noninvasive methods early in the disease process, such as in the mild cognitive impairment (MCI) stage or even the preclinical phase in which individuals are cognitively normal but have AD pathological changes, is crucial for the development of interventions to slow down cognitive decline or even prevent the onset of dementia.

Clinical studies have indicated that sleep is critical for the establishment of memories^8^. Studies have reported that memory establishment can be enhanced or impaired by directly modifying the sleep macroarchitecture or sleep electroencephalogram (EEG) microarchitecture^9,10^. Moreover, the aging process is known to be associated with a reduction in sleep time and with sleep fragmentation^11^. Sleep is critical for the metabolic clearance of Aβ in the brain^12^. Thus, there is growing interest in examining changes in sleep during the progression of dementia to further identify specific electrophysiological sleep features as possible early biomarkers of the neurodegenerative process^13^. In patients with AD and MCI, subjective and objective sleep disturbances are prevalent at levels as high as 40–60%^14,15^. Moreover, sleep disturbance in cognitively normal people is also correlated with a higher risk of AD later in life^16–19^. However, how these changes in sleep relate to cognitive deficits at the earlier stage of AD in both humans and animals is still unclear. In addition to sleep features, the role of the autonomic nervous system in the etiology of AD has received increasing attention in recent years^20^. Autonomic nervous system deterioration during dementia is believed to be due to the combined effects of aging and neurodegeneration in the prefrontal cortex^21^, telencephalic structures (anterior cingulate cortex, insula, and amygdala), hypothalamus, and brainstem^22^; these are crucial for not only autonomic modulation but also cognitive and emotional processes^22–26^. The neurodegenerative process also affects the neurotransmitter systems, especially the cholinergic system in AD, leading to reduced acetylcholine production^27^. By contrast, both animal and human studies have revealed that the storage of new information in memory can be enhanced by vagal nerve stimulation^28–31^, resulting in the release of acetylcholine^32^. Autonomic dysfunction may indeed be considered an early marker for dementia because degeneration in the insular cortex, the brainstem, and the cholinergic system occur early in the course of the disease—even before the emergence of overt clinical symptoms indicating the onset of dementia^33–35^. Heart rate variability (HRV) is calculated from the variations in the time intervals between successive heartbeats and its measurement constitutes a convenient and noninvasive means of assessing cardiac autonomic function. In particular, high-frequency HRV (HF-HRV) is a well-established indicator of vagal activity^36^, which is primarily modulated by the parasympathetic nervous system^37^. Higher resting HRV, especially HF-HRV, has frequently been linked to cognitive function^38,39^. However, the sleep–wake transition and the regulation of autonomic function have been reported to be intimately related^40,41^; the sympathetic tone is dominant during wakefulness, whereas the vagal tone is mostly dominant during sleep^42–44^. The majority of these studies have examined autonomic function during wakefulness; thus, the role of HRV in cognitive function is poorly understood in the context of sleep.

In the APP/PS1 double-transgenic mouse model of AD, Aβ aggregation begins to develop in the brain cortex at approximately 4 months of age; this aggregation increases in size and number with aging^45^. Deficits in spatial learning and memory performance as measured by the Morris water maze then emerge between 6 and 10 months and worsen with aging^45^. Therefore, the identification of biomarkers between the beginning of Aβ aggregation and the emergence of cognitive deficits in these mice may provide useful clues for the identification of early diagnostic markers in humans and further facilitate earlier interventions to prevent the occurrence of cognitive decline. Accordingly, the present study had 2 goals. The first was to investigate the differences in physiological biomarkers, including sleep patterns and autonomic function at different sleep–wake stages between APP/PS1 mice at the early stage (4 months of age; before marked cognitive impairment) and at the advanced stage (8 months of age; with cognitive impairment), and their age-matched wild-type (WT) littermates. The second was to further explore whether these physiological biomarkers are related to cognitive performance. We hypothesized that (1) sleep disturbance and sleep-related autonomic dysfunction would be more evident in APP/PS1 mice than in control mice at the early stage and that these phenomena would also be observed in APP/PS1 at the advanced stage and that (2) the severity of sleep disturbance or autonomic dysfunction would be significantly correlated with the presence of cognitive deficits.

## Methods

### Animal preparation

Male APP/PS1 transgenic mice (B6.Cg-Tg[APPswe, PSEN1dE9]85Dbo/Mmjax; No. 005864) were purchased from Jackson Laboratory (Bar Harbor, ME, USA) for breeding with female WT C57BL/6J mice. The APP/PS1 transgenic mice expressed a chimeric mouse–human APP695 that harbored Swedish K595N/M596L mutations (APPswe) and human PS1 with the exon-9 deletion mutation (PS1dE9), both of which were controlled by the mouse prion protein promoter, resulting in abundant amyloid plaques in the cortex and hippocampus^46^. Experiments were individually conducted on mice of 16–21 weeks of age (in the early stage of AD pathology) and 32–37 weeks of age (in the advanced stage of AD pathology); these mice were male APP/PS1 transgenic mice and their sex- and age-matched WT littermates. We used the resource equation approach^47^ to calculate the required sample size for each group in this study, which ranged from 14 to 24 mice. The number of mice at each experimental stage was adjusted based on different exclusion criteria (Table S1). The mice were raised in a sound-attenuated room with a 12:12-h light–dark cycle (09:00–21:00, light on). In this study, we considered Zeitgeber time (ZT) 0 to represent light on and ZT 12 to be light off. The mice were provided with food and water ad libitum and were housed under controlled room temperature (22 ± 2 °C) and humidity (40–70%). Additionally, we provided nesting material to enhance the environmental enrichment for the mice. The experimental procedures were approved by the National Yang Ming Chiao Tung University’s Institutional Animal Care and Use Committee. All animal experiments were conducted in accordance with ARRIVE guidelines. All methods were performed in accordance with relevant guidelines and regulations.

### Surgical procedures

All mice underwent surgery for physiological signal collection. The surgery was performed on 16-week-old and 32-week-old mice individually in the following manner. Under pentobarbital anesthesia (50–60 mg/kg, intraperitoneal injection), the mouse was placed on a standard stereotaxic apparatus. The skull surface was exposed, 3 screws were fixed into it, 2 stainless steel microwires served as the right frontal electrode (1.0 mm anterior to and 1.0 mm lateral to the bregma), and another stainless steel wire was fixed in the occipital bone acted as the reference electrode (1.0 mm caudal to the lambda). The electrocardiogram (ECG) signal was recorded with a pair of microwires placed under the dorsal skin (one between the cervical and thoracic levels of the left lateral side of the chest wall, and the other at the lumbar level of the right lateral side of the abdominal wall), and the electromyogram (EMG) signal was recorded by two seven-strand stainless steel microwires bilaterally inserted into the dorsal neck muscles^48^. All the electrophysiological signals were joined to a connector (0.35 g) fixed to the skull by dental acrylic (1.25 g) (Fig. 3A and B). After surgery, the mice were treated with an antibiotic (cephalexin hydrate, 15 mg/kg, subcutaneous injection) and an analgesic (carprofen, 5 mg/kg, subcutaneous injection), and housed individually for at least 7 days for recovery.

### Physiological signal acquisition

A KY1C wireless sensor (23 × 14 × 13 mm^3^ and 4 g), consisting of a transmitter and battery, from K&Y Lab (Taipei, Taiwan) was used to record the electrophysiological signals (Fig. 3C). Based on previous literature, a wireless system should not exceed approximately 5 g in total weight to allow truly unconstrained movement of the mouse. Additionally, rodents can readily carry about 10–15% of their body weight^49^. The sensor used in this study weighing 4 g is within the recommended weight limit. Moreover, we measured the body weight of the mice before recording physiological signals to ensure that the 4 g sensor does not impact their behavior. For the 18–19-week-old WT and APP/PS1 mice, the body weight ranges were 27–31 g. For the 34–35-week-old WT and APP/PS1 mice, the body weight ranges were 30–33 g and 30–34 g, respectively. The weight of the 4 g sensor is less than 15% of the minimum body weight (27 g), which calculates to 4.05 g. Furthermore, based on our observations, mice carrying the sensor were able to perform all normal behaviors, including running, exploring, and grooming. Consequently, the sensor used in this study did not affect animal behavior and recorded physiological signals of freely-moving mice. The data acquisition and storage system used with the mice was modified from that used with rats^41,44^; the system’s performance was validated in a previous study^48^. The EEG and EMG signals were amplified 1000-fold, and the ECG signal was amplified 500-fold. The EEG signal was band-pass filtered at 0.16–53.05 Hz, and the EMG and ECG signals were filtered at 0.72–112.88 Hz. The EEG, EMG, and ECG signals were synchronously digitized using a 12-bit analog–digital converter at different sampling rates (125, 500, and 500 Hz, respectively). The digitized dataset was stored on a flash memory card for subsequent offline analysis.

### Experimental protocols

The experiments began more than 1 week after surgery. To make the mice habituate to the experimental apparatus, the mice were temporarily and physically restrained to allow the wireless sensor to be attached to the connector on their head for at least one day. After the habituation period, when the light was turned on, we started recording physiological signals of mice by remotely activating the sensors from the computer software without directly contacting the mice. Due to the limited battery capacity of the wireless sensor, we first took measurements for 5.5 h with one sensor (ZT 0–5.5). At the end of the first recording period (ZT 5.5), we immediately replaced the sensor, allowing the mice to calm down and rest for at least 20 min to reduce the stress associated with changing the sensor before continuing the physiological signal recording (ZT 6–11.5). Thus, a total of 11 h of physiological signals were recorded during the light period. Physiological signals were recorded in mice of 18–19 and 34–35 weeks of age individually. After physiological recording, the cognitive functions in mice at 20–21 and 36–37 weeks of age were investigated with behavioral tests, namely novel object recognition and the Morris water maze. During the testing phase, the wireless sensor was not attached to the connector on the head of the mouse. All mice were sacrificed with an overdose of pentobarbital (100 mg/kg, intraperitoneal injection) after the behavioral tests. Histochemistry of the brain sections was analyzed for Aβ accumulation and glial fibrillary acidic protein (GFAP) levels associated with inflammation.

### Sleep and EEG frequency analysis

The procedure for sleep pattern analysis was modified from that described in previous reports for mice^50^. For the EEG and EMG signals, continuous power spectral analysis was performed with a 16-s Hamming window (50% overlap). Our algorithm estimated the power density of the spectral components based on the fast Fourier transform. The resulting power spectrum was corrected for the attenuation caused by sampling and the application of the Hamming window^51^. For each 16-s time segment, we quantified the frequency components of the EEG spectrogram (sigma power, 10–14 Hz; theta power, 6–9 Hz; delta power, 0.5–4 Hz)^50^, and the EMG spectrogram (32–64 Hz). The conscious state for each time epoch (8 s) was defined as active waking (AW), quiet sleep [QS; also called non-rapid eye movement (NREM) sleep], or paradoxical sleep (PS; also called REM sleep) according to the following variable parameters: the product of sigma power and theta power (sigma*theta), the delta power, and the EMG power. AW states were characterized by high EMG activity and low sigma*theta, whereas sleep (QS and PS) was characterized by low EMG and high sigma*theta. In a subdivision of sleep states, sleep states with high delta activity were identified as QS, whereas those with low delta activity were identified as PS. AW was distinguished from sleep by the slope and intercept values of a separation line on the plot of sigma*theta versus EMG, and QS was distinguished from PS with a delta threshold. The slope and intercept values of the separation line and delta threshold were adjusted via visual inspection by one blinded experimenter. The adjusted values were presented in Table S2. There were no significant differences observed between different mouse strains or between different disease stages. One sleep–wake stage comprised at least 6 consecutive identical epochs (~ 56 s), and interruptions were defined as a period comprising fewer than 6 consecutive epochs. Stage time (total time spent in a specific stage during analysis), bout number (number of bouts for a specific stage), and bout duration (average bout duration for a particular stage) were calculated to quantify sleep–wake stage structure. Moreover, the delta power percentage^52^ and interruptions during QS were used to assess sleep quality.

### HRV analysis

The digital processing of the bioelectric signals was similar to the procedures described in our previous study^48^. The computer algorithm identified each normal ventricular discharge waveform and rejected any ventricular premature complex or noise according to its likelihood relative to a standard template. To provide continuity in the time domain, stationary R–R intervals (RR) were resampled and interpolated at a rate of 64 Hz before being truncated into successive 16-s (1024 points) time segments (windows) with a 50% overlap. A Hamming window was applied to every time segment to attenuate the leakage effect. Our algorithm then estimated the power density of the spectral components based on the fast Fourier transform. The resulting power spectrum was corrected for the attenuation caused by sampling and the application of the Hamming window. For each 16-s time segment, we quantified the RR interval duration and the frequency components of the RR spectrogram (total power [TP] not including frequency components below 0.4 Hz, high-frequency power [HF] at 1.5–4.0 Hz, low-frequency power [LF] at 0.4–1.5 Hz, and LF% defined as LF/TP)^53^. TP, HF, and LF% indicated the overall cardiac autonomic activity, cardiac vagal activity, and cardiac sympathetic modulation, respectively. LF/HF indicated sympathovagal balance where increases in LF/HF are assumed to reflect a shift to “sympathetic dominance” and decreases in this index correspond to a “parasympathetic dominance”^36,44^.

### Novel object recognition

#### Apparatus

Experiments were performed in a white plexiglass box (40 × 40 × 40 cm^3^). A video camera was positioned over the arena, and sessions were videotaped for later analysis. Two identical objects were used as samples, and a different novel object was used for discrimination. The objects used included plastic rectangular blocks (4.8 × 2.4 × 6 cm^3^) composed of four different colored small blocks (yellow, red, green, and blue), as well as a plastic blue opaque sphere with a diameter of 5.6 cm. Both objects had smooth surfaces that were difficult to be gnawed by mice and easy to clean. Moreover, the objects were affixed to the floor to ensure that the mice could not displace them. Objects and the open field were cleaned thoroughly between trials with 70% ethanol solution.

#### Behavioral procedures

The mice were first habituated to the open field by being placed individually in the field for 10 min. One day after habituation, each mouse underwent a test session comprising a sample phase, a delay period of 60 min, and the discrimination phase. In the sample phase, mice were exposed to 2 identical objects (A1 and A2) for 10 min. These objects were placed in 2 opposite corners of the open-field arena. After the end of the sample phase, the mice were placed in their home cages. After a delay period of 60 min, an identical copy of the familiar object (A3) and a novel object (B) were placed in the locations previously occupied by A1 and A2 in a randomized and counterbalanced order. Also, we counterbalanced the use of each set of objects so that each object was used equally as a familiar object and as a novel object. In the discrimination phase, the mice were allowed to explore A3 and B for 5 min in a randomized order. The experiment was conducted by one blinded experimenter. Next, the time spent exploring each object was assessed from the videotape recording of each session. Exploration of an object was defined as the mouse directing its nose to the object at a distance of less than 2 cm; touching it with its nose, mouth, or paw; or both. Behavior was scored by 2 investigators who were blinded to the WT and AD mice types. For each test session, we measured the discrimination ratio [*Time*_novel_/(*Time*_novel_ + *Time*_sample_)], indicating the proportion of total object exploration time that was spent investigating the novel object during the discrimination phase. The task was performed to evaluate deficits in recognition memory^54^.

### Morris water maze

#### Apparatus

A white plastic circular swimming pool (1.2 m in diameter) was filled with water maintained at 22 ± 2 °C. Room cues visible from the water surface were constant from day to day. A clear plexiglass escape platform (10 cm in diameter) was positioned at the center of one of the quadrants at 1 cm below the water surface. Additionally, during the experiment, we confirmed daily that the platform was positioned 1 cm below the water surface to prevent difficulties for the mice in climbing onto it.

#### Behavioral procedures

Prior to the experiment, the mice were not trained to swim or climb onto the platform. However, we allowed the mice to become familiar with the experimental space containing the swimming pool for at least 3 h to reduce the stress caused by transportation to a different experimental environment and unfamiliarity with the surroundings. The water maze spatial learning test occurred over a 6-day period. The first 5 days were the acquisition (i.e., training) days. On the sixth day, the probe (or retention) trial was performed. The training period comprised four 60-s trials with 15-min intertrial intervals per day. The mouse began at 4 different positions in the water maze in a randomized and counterbalanced order. For the training trials, the time taken to reach the invisible platform was recorded. On the 6th day, a probe trial of 60 s was performed after the escape platform had been removed. The release position of the probe differed from the 4 positions used for the spatial trials during the previous 5 testing days. For the probe trial, the number of platform crossings was recorded and analyzed with the EthoVision XT video tracking system (Version 13, Noldus, Wageningen, Netherlands). The testing procedure provides a measure of spatial learning and memory ability^55^.

### Histochemistry

The mice were perfused with 4% formaldehyde, and their brain tissue was cryoprotected. Free-floating 30-µm coronal brain sections (− 1.34 to − 1.58 mm relative to the bregma) were stained with 0.01% (trans, trans),-1-bromo-2,5-bis-(3-hydroxycarbonyl-4-hydroxy) styrylbenzene (BSB) as previously described^56^ to detect amyloid plaques and were then washed in PBS with Tween-20. To detect the astrocyte activation in the vicinity of amyloid plaques, the brain slices were incubated with mouse anti-GFAP antibody (1:5000, Sigma) at 4 °C overnight. After washing, the brain slices were incubated with donkey anti-mouse Alexa Fluor 594 secondary antibody (1:500, Alexa) and counterstained with 4′,6-diamidino-2-phenylindole (DAPI) (1:1000, Sigma-Aldrich). After the second wash, the sections were mounted in Aqua Poly/Mount (Polyscience, Warrington, PA, USA), and images were captured with a Zeiss Axioplan 2 microscope. Two serial sections were analyzed for each mouse. The coverage of senile plaques and GFAP in cortex were quantified using ImageJ software.

### Statistical analysis

The spectral power of the EEG spectrogram and the TP, LF, and HF of the RR spectrogram were logarithmically transformed to correct for their skewed distributions^51^. Normality was formally tested using the Shapiro–Wilk test for all variables. For comparisons of 2 unpaired groups (WT vs. APP/PS1; early-stage AD pathology vs. advanced-stage AD pathology), the independent *t* test was used if the normality hypothesis was not rejected by the Shapiro–Wilk test; otherwise, preference was given to the Mann–Whitney *U* test. To compare the time taken by mice to reach the platform during the first five days of the Morris water maze, Fisher’s least significant difference test was performed following a significant one-way analysis of variance with repeated measures if normality was not rejected after the Shapiro–Wilk test; otherwise, Dunn’s post hoc method following a significant Friedman’s test was preferred^57^. Correlation between 2 parameters was assessed using linear regression, Pearson correlation analysis was used for normally distributed data, and Spearman correlation analysis was used for nonnormally distributed data. All statistical analyses were performed using SPSS (Version 22, IBM, Chicago, IL, USA). Statistical significance was considered *p* < 0.05. The results are presented as the mean ± SEM.

## Results

### APP/PS1 mice exhibited amyloid plaque burden and neuroinflammation but no cognitive impairments at the early stage of the disease

**Figure 1:**
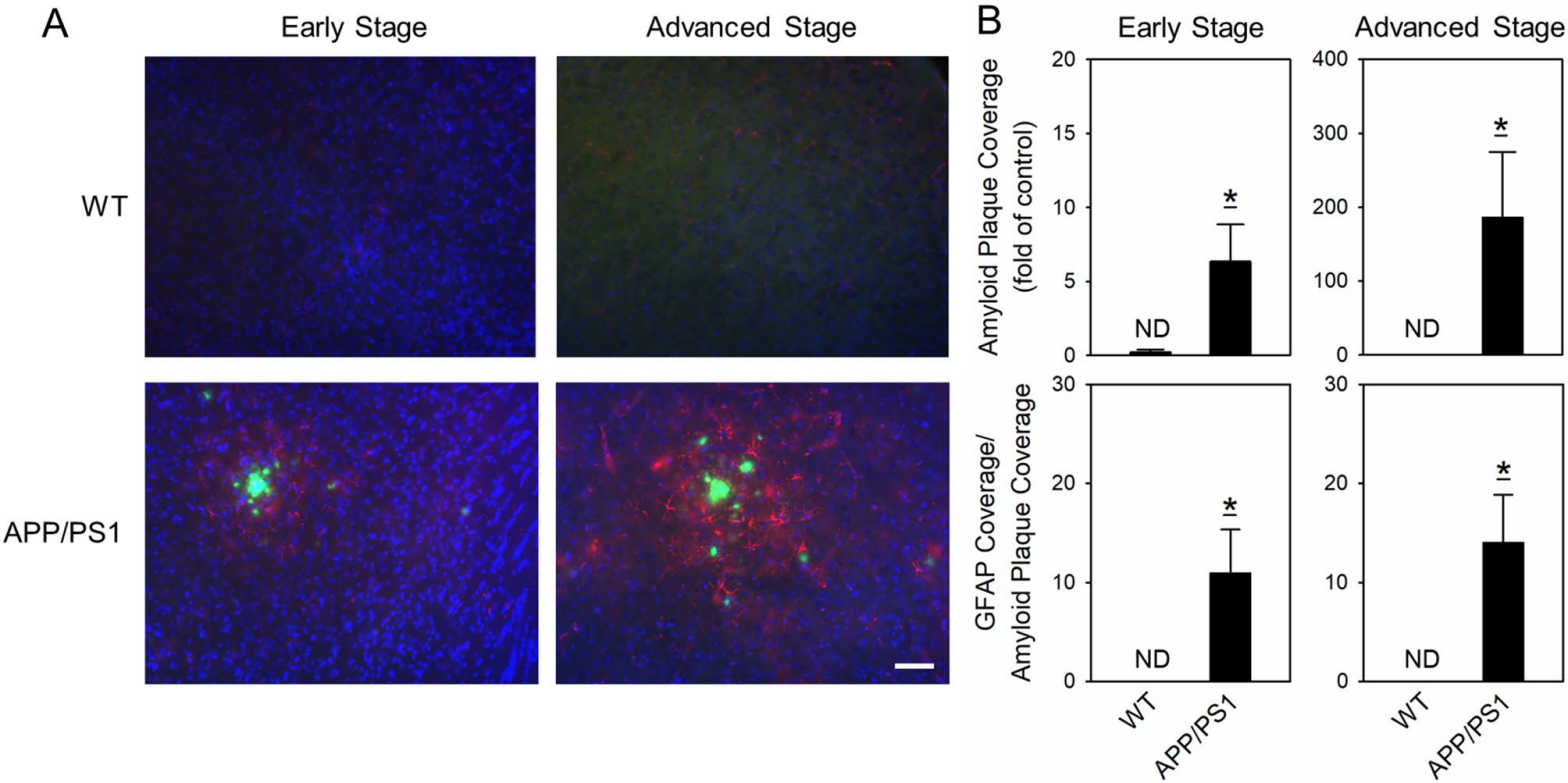
Amyloid plaques and activation of astrocytes in APP/PS1 mice individually at the early (21 weeks of age) and advanced (37 weeks of age) stages of the disease and their age-matched WT littermates. (**A**) Representative immunohistochemical images of amyloid plaques (green), GFAP (red), and DAPI (blue) in the cortices of 21- and 37-week-old WT and APP/PS1 mice. Scale bar, 50 μm. (**B**) Histograms for the amyloid plaque coverage and GFAP coverage per amyloid plaque coverage in 21- and 37-week-old WT and APP/PS1 mice. ND is non-detectable. Values are presented as mean ± SEM. Early stage: WT, n = 9; APP/PS1, n = 10; advanced stage: WT, n = 7, APP/PS1, n = 6. *p < 0.05 vs WT mice by Mann–Whitney *U* test for nonnormality. DAPI, 4′,6-diamidino-2-phenylindole; GFAP, glial fibrillary acidic protein; WT, wild type.

To assess the AD pathogenesis, neuropathological characteristics and cognitive behaviors were compared between WT and APP/PS1 mice at the early (20–21 weeks of age) and the advanced (36–37 weeks of age) stages of the disease. Amyloid plaques are one of the main neuropathological biomarkers of AD and can induce neuroinflammatory activation of glia. BSB amyloid staining reagent and anti-GFAP antibody were used to detect amyloid plaques and reactive astrocytes, respectively. Activated astrocytes observed in the fluorescent images had clustered at the foci of the amyloid plaques in the cortices of early- and advanced-stage APP/PS1 mice. Low GFAP was expressed and no amyloid plaques were detected in the cortices of their age-matched WT littermates (Fig. 1A). The coverages of amyloid plaques and the ratio of GFAP coverage to amyloid plaque coverage were significantly higher in early- and advanced-stage APP/PS1 mice than in their age-matched WT littermates (*p* < 0.05 for both; Fig. 1B).

**Figure 2:**
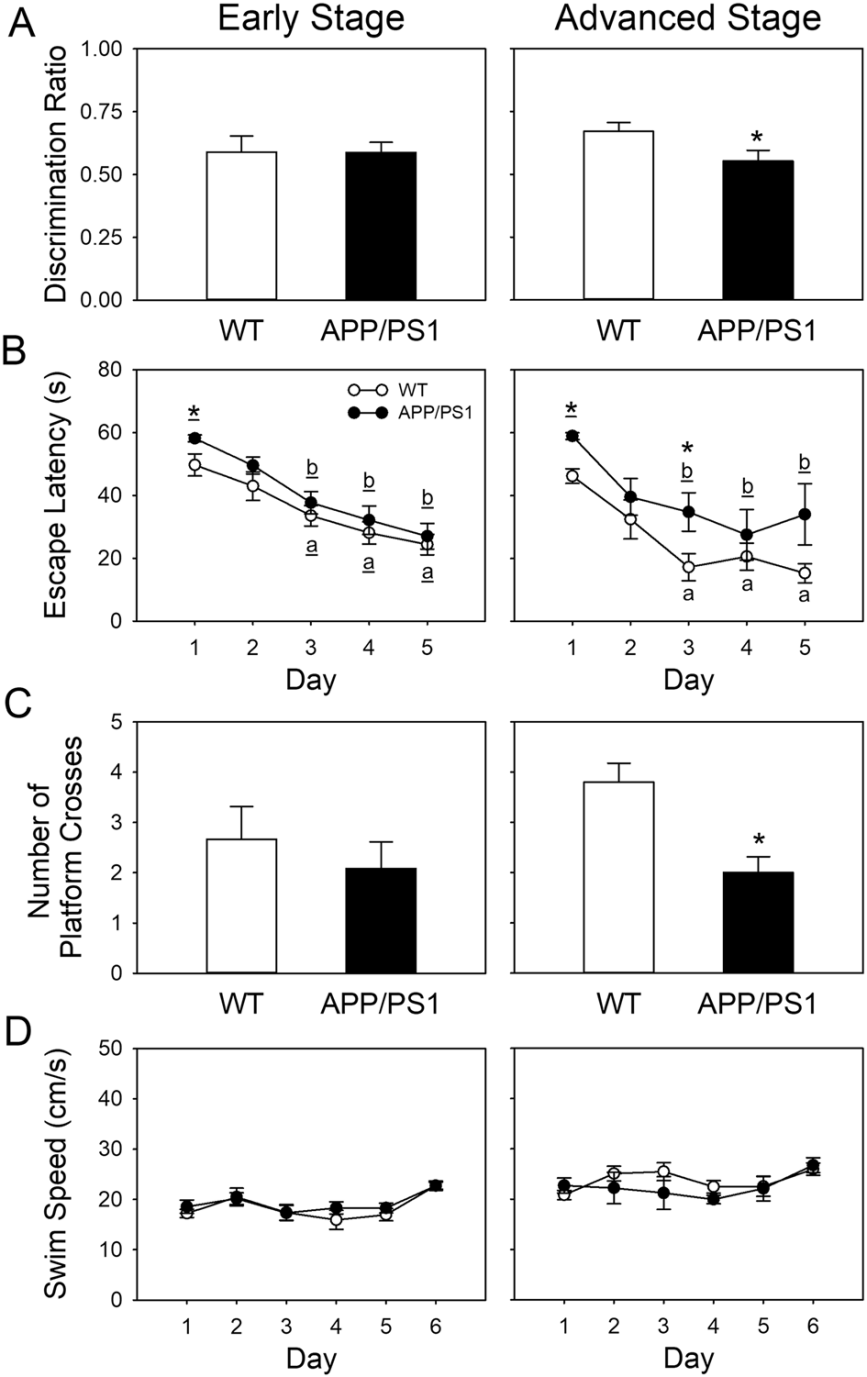
Cognitive function in APP/PS1 mice individually at the early (20–21 weeks of age) and advanced (36–37 weeks of age) stages of the disease and their age-matched WT littermates. (**A**) Recognition memory by the indexes of the novel object recognition task of discrimination ratio. Early stage: WT, n = 9; APP/PS1, n = 10; advanced stage: WT, n = 10; APP/PS1, n = 9. (**B** and **C**) Spatial learning and memory ability indicated by indices for the Morris water maze task of (**B**) average escape latency for finding the platform (seconds) and (**C**) the number of times that the mice crossed the original platform location in the probe trial. (**D**) Average swimming speed during the 6 consecutive days of the Morris water maze. Early stage: WT, n = 9; APP/PS1, n = 12; advanced stage: WT, n = 5, APP/PS1, n = 5. The results are expressed as means ± SEM. *p < 0.05 vs. WT by independent *t* test, *p < 0.05 vs. WT by Mann–Whitney *U* test. ^a^*p* < 0.05 vs. the first day of the Morris water maze in WT by Fisher’s least significant difference test following a significant one-way repeated-measures analysis of variance (ANOVA), ^a^*p* < 0.05 vs. the first day of the Morris water maze in WT by Dunn post-hoc method following a significant Friedman test. ^b^*p* < 0.05 vs. the first day of the Morris water maze in APP/PS1 by Dunn post-hoc method following a significant Friedman test. WT, wild type.

The results of the novel object recognition and the Morris water maze tasks were used to evaluate recognition memory and spatial learning and memory, respectively. In the novel object recognition task, the discrimination ratio of early-stage APP/PS1 mice was not significantly different from that of WT mice, and advanced-stage APP/PS1 mice had a significant reduction (*p* = 0.048) in their discrimination ratio relative to WT mice (Fig. 2A). During the first 5 days of the Morris water maze, both WT and APP/PS1 mice in the early and advanced stages showed a significant decrease in the time taken to locate the platform from the third day to the fifth day, compared to the first day (*p* < 0.05 for both). When comparing WT and APP/PS1 mice, early-stage APP/PS1 mice spent more time locating the platform on the first day compared with age-matched WT mice (*p* = 0.023), and advanced-stage APP/PS1 mice took significantly more time to reach the platform on not only the first day (*p* = 0.008) but also on the third day (*p* = 0.047) (Fig. 2B). On the 6th day of the probe trial, the number of platform crosses of early-age APP/PS1 mice were not significantly different compared with their WT littermates. By contrast, advanced-stage APP/PS1 had fewer platform (*p* = 0.006) crosses than WT mice did (Fig. 2C). Moreover, there were no significant differences observed in swimming speed when comparing between genotypes (Fig. 2D).

### APP/PS1 mice had sleep problems and autonomic dysregulation at the early stage of the disease

**Figure 3:**
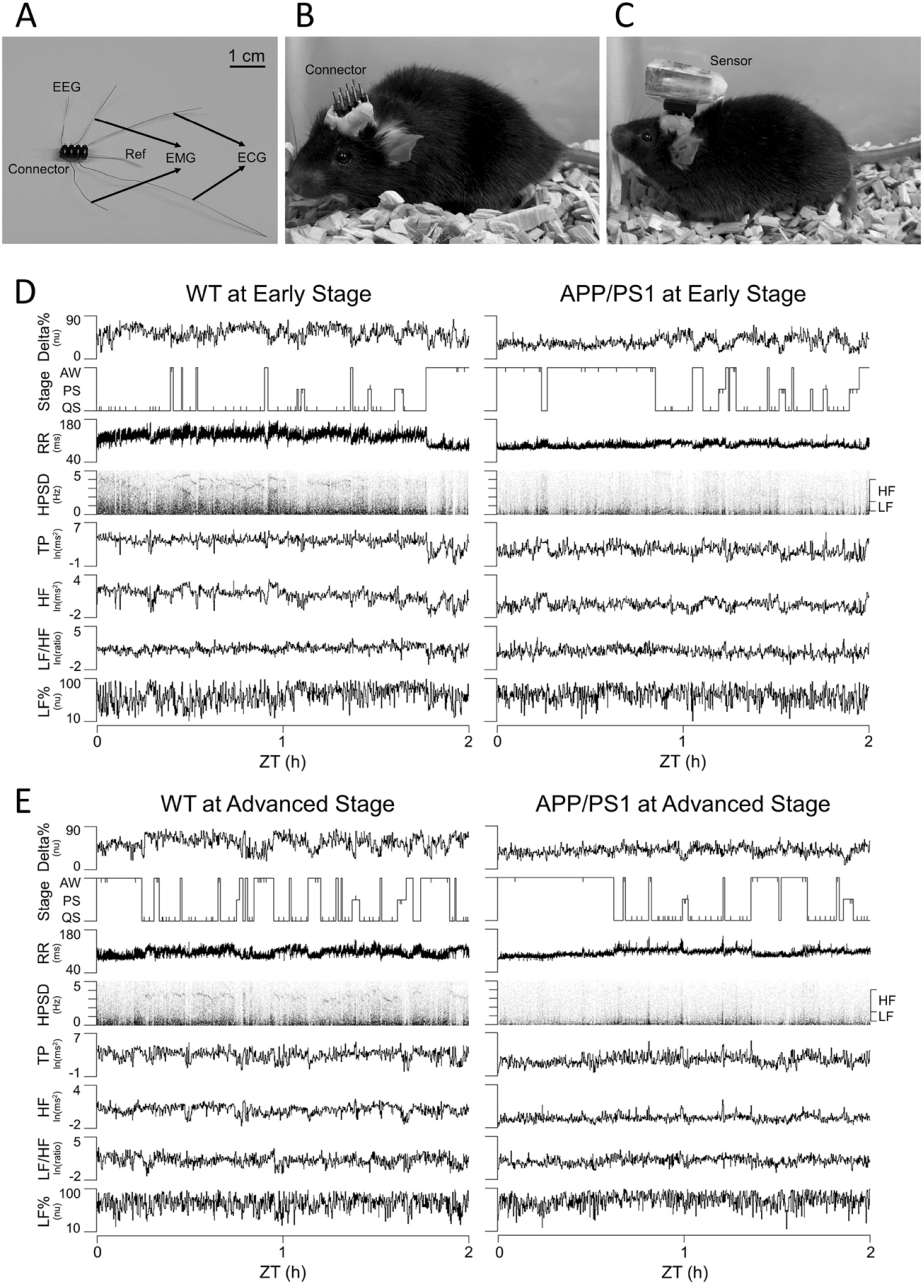
Physiological signals acquisition by using the wireless sensor and continuous and simultaneous analyses of various physiological signals in APP/PS1 mice at the early (18–19 weeks of age) and advanced (34–35 weeks of age) stages of the disease and in their age-matched wild-type (WT) littermates. (**A**) Handmade connector with electroencephalogram (EEG), electromyogram (EMG), and electrocardiogram (ECG) electrodes. (**B**) The connector on the head of the mouse. (**C**) The wireless sensor was attached to the connector on the head of the mouse. (**D** and **E**) Continuous and simultaneous analyses of delta power percentage of the electroencephalographic spectrogram (Delta%), sleep–wake state, and heart rate variability during the first 2 h (ZT 0–2) of the light period for a WT mouse and an APP/PS1 mouse at (**D**) the early stage (18–19 weeks old) and (**E**) the advanced stage (34–35 weeks old) of the disease. The sleep stages (Stage) included active waking (AW), paradoxical sleep (PS), and quiet sleep (QS). Interruptions of QS were marked by the vertical ticks in the hypnogram. R–R interval (RR) and its power spectrogram (HPSD) are displayed with temporal alterations in total power (TP), high-frequency power (HF), low-frequency power to high-frequency power ratio (LF/HF), and normalized low-frequency power (LF%) of heart rate variability. The range of frequencies for HF and LF are denoted on the right side of the spectrograms. Ref, reference; ln, natural logarithm; nu, normalized units; ZT, Zeitgeber time.

To explore the physiological parameters in WT and APP/PS1 mice before the assessments of cognitive behaviors and neuropathology, we continuously and simultaneously recorded various physiological signals for 11 h during the light period by using the wireless sensor when the mice were 18–19 (early stage) and 34–35 weeks old (advanced stage) (Fig. 3A, B and C). Continuous analysis of the EEG power, sleep architecture, and HRV of the first 2 h (ZT 0–2) during the light period are presented in Fig. 3D and E. The hypnogram clearly revealed that sleep decreased remarkably in APP/PS1 mice at the early stage (Fig. 3D) but not at the advanced stage (Fig. 3E) compared with their age-matched WT littermates. Compared with age-matched WT mice, delta power%, RR, TP, and HF notably declined in both early- (Fig. 3D) and advanced-stage APP/PS1 mice (Fig. 3E).

**Figure 4:**
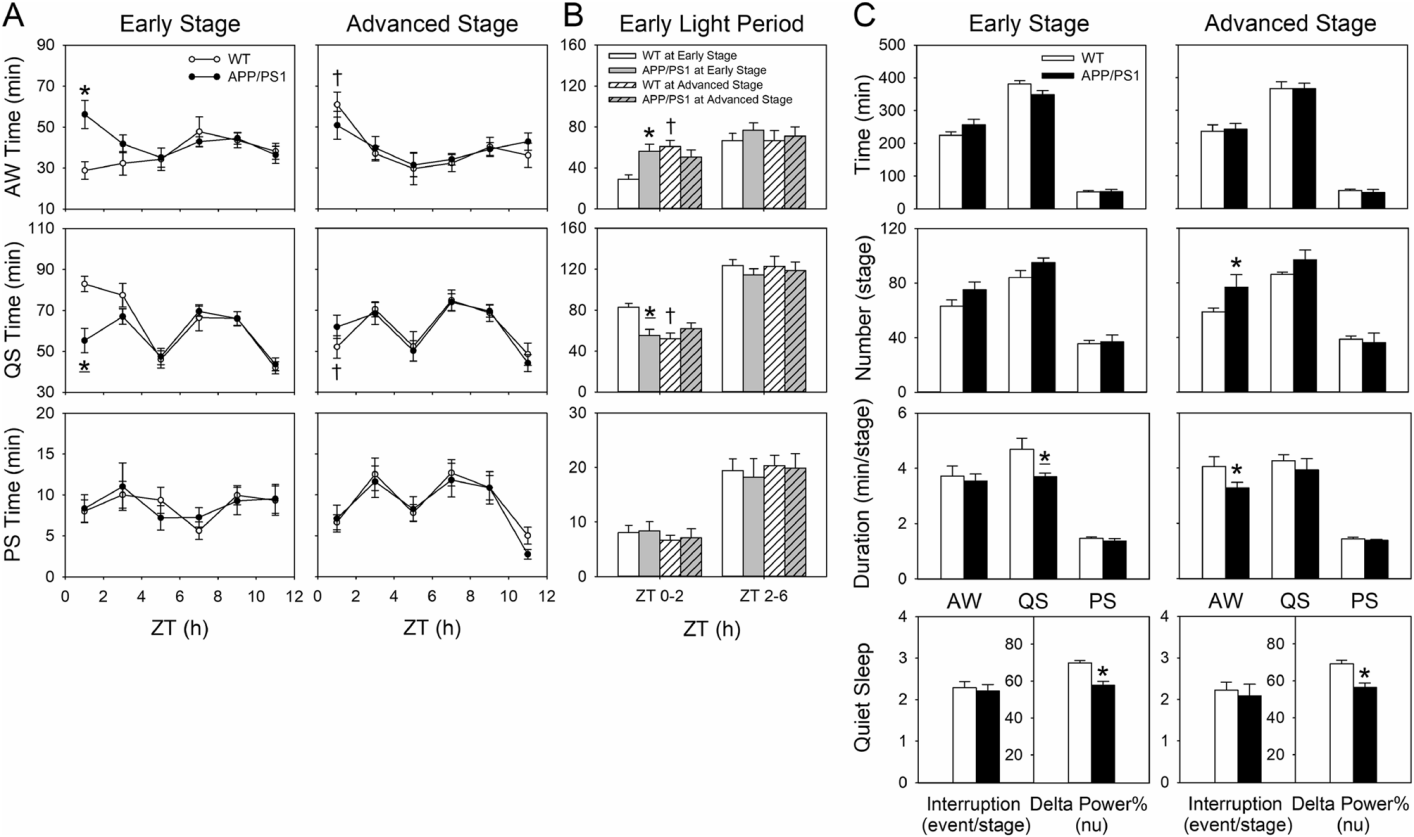
Sleep patterns during 11 h of the light period in APP/PS1 mice individually at the early (18–19 weeks of age) and advanced (34–35 weeks of age) stages of the disease and in their age-matched WT littermates. (**A** and **B**) Accumulated AW, QS, and PS times using a 2-h window (**A**) during the light period over 11 h and (**B**) during ZT 0–2 and ZT 2–6 of the early light period over 5.5 h. Due to the 5.5-h battery life limit of the wireless sensor, we only analyzed the 1.5 h of data recorded between ZT 4–6 and ZT 10–12. (**C**) Time, number, and duration during different sleep–wake stages and interruptions and delta power% of the electroencephalographic spectrogram during the QS stage. Values are presented as mean ± SEM. Early stage: WT, n = 7; APP/PS1, n = 11; advanced stage: WT, n = 8, APP/PS1, n = 8. *p < 0.05 vs. WT by independent *t* test, *p < 0.05 vs. WT by Mann–Whitney *U* test. ^†^*p* < 0.05 vs. early stage in WT by independent *t* test, there were no significant differences between the early stage and advanced stage of the disease in APP/PS1 mice by either independent *t* test or Mann–Whitney *U* test. AW, active waking; QS, quiet sleep; PS, paradoxical sleep; Time, total time spent in a specific stage within the analysis period; Number, number of bouts for a specific stage; Duration, average duration of bouts for a particular stage; nu, normalized units; WT, wild type.

We investigated sleep architecture changes during the AW, QS, and PS stages using 2-h (Fig. 4A and B) and 11-h windows (Fig. 4C). The early-stage APP/PS1 mice spent more time in AW (*p* = 0.010) and less time in QS (*p* = 0.008), mainly during ZT 0–2, than did WT mice, and no significant differences in accumulated PS time were observed between them. By contrast, no significant differences in accumulated time were observed at each sleep stage between advanced-stage APP/PS1 and WT mice (Fig. 4A). Further, we focused on exploring changes in sleep during the early light period (ZT 0–6) between mice at early and advanced stages of the disease because sleep time during the late light period (ZT 6–12) in APP/PS1 mice did not differ from that in WT mice at both stages of the disease (Fig. 4B). WT mice at the advanced stage spent more time in AW (*p* = 0.001) and less time in QS (*p* = 0.001) during ZT 0–2 compared with early-stage mice; no significant differences were observed between early-stage and advanced-stage APP/PS1 mice (Fig. 4A and B). However, a quantitative analysis of the sleep architecture changes using 11-h windows revealed no significant differences between early-stage APP/PS1 mice and WT mice in accumulated time and stage number during different sleep–wake stages, but the early-stage APP/PS1 mice had shorter QS stage durations than did WT mice (*p* = 0.004). At the advanced stage, APP/PS1 mice also had no significant changes in accumulated time at each sleep stage; however, they had a higher frequency (*p* = 0.047) and shorter duration (*p* = 0.048) in the AW stage compared with WT mice. Moreover, interruptions of QS did not significantly differ between either early-stage or advanced-stage APP/PS1 mice and their age-matched WT littermates; however, APP/PS1 mice at both stages had lower delta power% (an indicator of sleep depth) than their WT counterparts (*p* < 0.05 for both). Additionally, with 11-h windows, neither WT nor APP/PS1 mice had significant changes in sleep architecture between the early and advanced stages (Fig. 4C).

**Figure 5:**
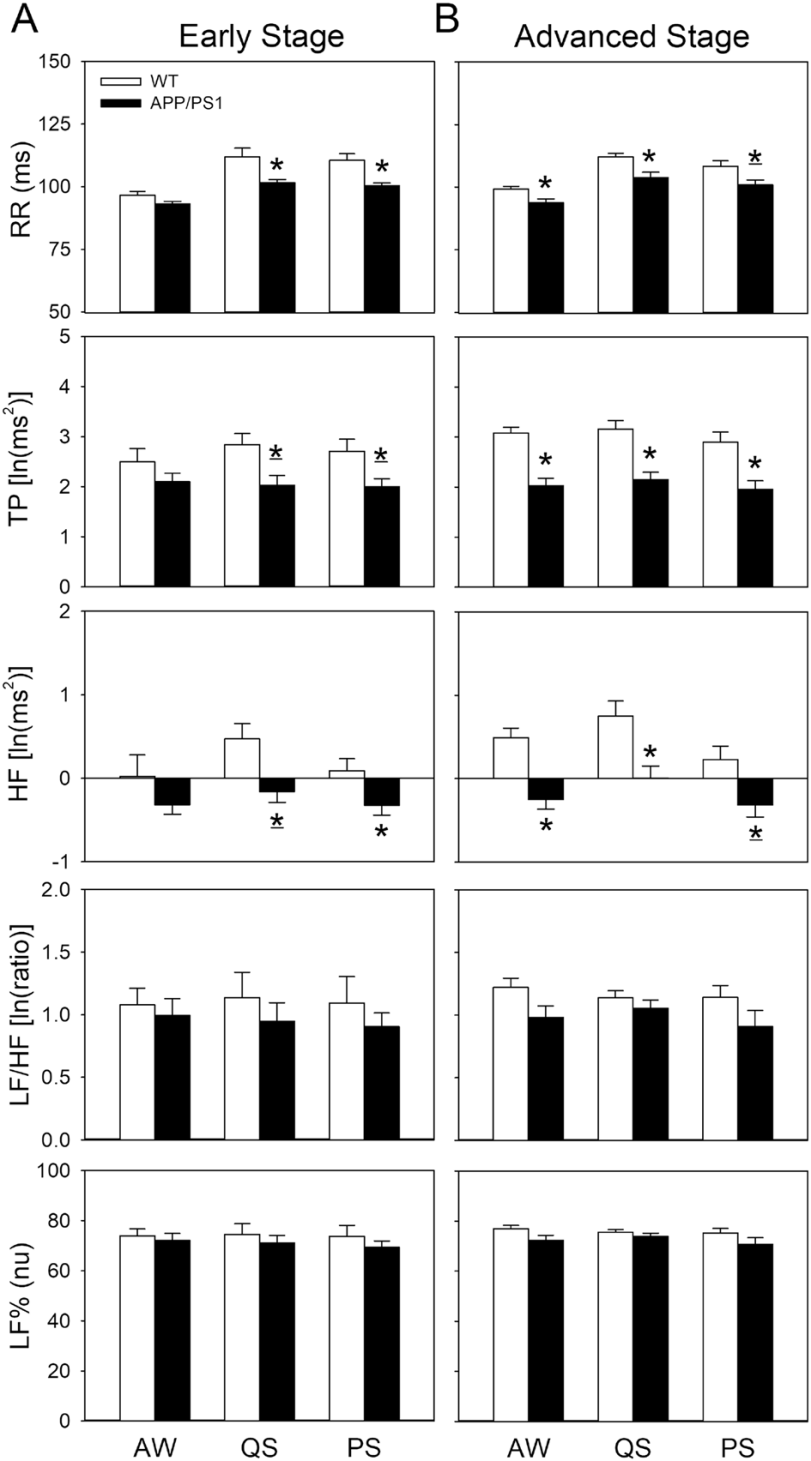
Cardiac autonomic function during different sleep–wake states during the light period in WT and APP/PS1 mice individually at (**A**) the early (18–19 weeks of age) and (**B**) advanced (34–35 weeks of age) stages of the disease. Values are presented as mean ± SEM. Early stage: WT, n = 7; APP/PS1, n = 11; advanced stage: WT, n = 8, APP/PS1, n = 8. *p < 0.05 vs. WT by independent *t* test, *p < 0.05 vs. WT by Mann–Whitney *U* test. There were no significant differences between the early stage and advanced stage of the disease by independent *t* test or Mann–Whitney *U* test. AW, active waking; QS, quiet sleep; PS, paradoxical sleep; RR, R–R interval; TP, total power of heart rate variability; HF, high-frequency power of heart rate variability; LF/HF, low-frequency power to high-frequency power ratio of heart rate variability; LF%, normalized low-frequency power of heart rate variability; ln, natural logarithm; nu, normalized units; WT, wild type.

In addition to sleep patterns, we explored the differences in autonomic function during different sleep–wake stages in WT and APP/PS1 mice (Fig. 5). At the early stage of the disease, the mean RR, TP, and HF were significantly lower in APP/PS1 mice than in WT mice during sleep stages (*p* < 0.05 for both QS and PS). By contrast, no significant differences in LF/HF or LF% were observed between the groups (Fig. 5A). The advanced-stage APP/PS1 mice also had lower RR, TP, and HF during not only sleep stages but also during the AW stage compared with WT mice (*p* < 0.05 for all sleep stages). LF/HF and LF% did not differ from each other but had a tendency to decline in advanced-stage APP/PS1 mice compared with WT mice during the AW (LF/HF, *p* = 0.077; LF%, *p* = 0.086) and PS stages (LF/HF, *p* = 0.064; LF%, *p* = 0.068) (Fig. 5B). No significant differences in either WT or APP/PS1 mice were observed in comparisons of mice at the early stage and the advanced stage (Fig. 5).

To determine whether the interaction between sleep and the autonomic nervous system existed in either early- or advanced-stage mice, we analyzed the correlations between sleep parameters (time, interruption, and delta power%) and cardiac autonomic parameters (HF, LF/HF, and LF%) during light QS across all mice individually at the early or the advanced stage of the disease (Table 1). No significant correlations of accumulated time or interruption with each cardiac autonomic parameter were observed; however, delta power% had a strong positive correlation with HF but not with LF/HF or LF% during light QS at both early (r = 0.646, *p* = 0.004) and advanced stages (r = 0.672, *p* = 0.004) of the disease (Table 1). Thus, the correlation between delta power% and HF was further investigated in each group separately. Delta power% had a significant positive correlation with HF only in early-stage WT mice (r = 0.833, *p* = 0.020) but not in early-stage APP/PS1 or advanced-stage WT and APP/PS1 mice (Fig. S1).

**Table 1:** Correlation between sleep patterns and autonomic function variables during light quiet sleep across all mice individually at the early and advanced stages of the disease.

Variable	Time (min)		Interruption (event/stage)		Delta power% (nu)	
	r	*p*	r	*p*	r	*p*
Early stage (n = 18)						
HF [In(ms^2^)]	0.403	0.098	0.035	0.889	0.646*	0.004
LF/HF [In(ratio)]	0.133	0.598	− 0.060	0.812	0.356	0.147
LF% (nu)	0.137	0.587	− 0.010	0.967	0.330	0.181
Advanced stage (n = 16)						
HF [In(ms^2^)]	− 0.326	0.219	0.372	0.155	0.672*	0.004
LF/HF [In(ratio)]	0.347	0.188	0.379	0.148	0.282	0.290
LF% (nu)	0.329	0.213	0.376	0.151	0.286	0.282

^*^p < 0.05 in Pearson correlation analysis.

HF, high-frequency power of heart rate variability; LF/HF, low-frequency power to high-frequency power ratio of heart rate variability; LF%, normalized low-frequency power of heart rate variability; ln, natural logarithm; nu, normalized units; Time, accumulated time spent in the quiet sleep stage; Delta power%, relative delta power of the electroencephalographic spectrogram during the quiet sleep stage (also known as slow wave activity, an indicator of sleep depth).

### Autonomic function and sleep quality were correlated with cognitive function

**Figure 6:**
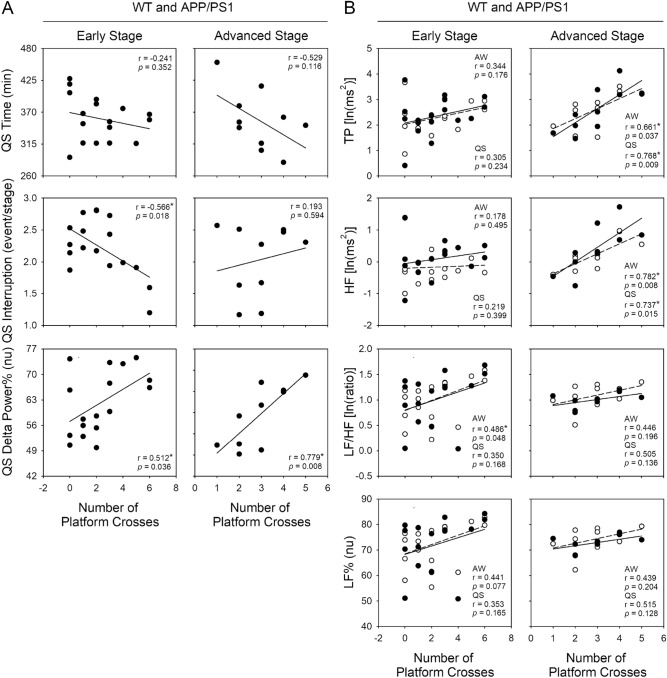
Relationship between physiological parameters and cognitive function in all mice (WT and APP/PS1 mice) individually at the early and advanced stages of the disease. (**A**) Correlations of accumulated time, interruption and delta power% of the electroencephalographic spectrogram in QS during the light period with the number of platform crosses. (**B**) The correlation of cardiac autonomic function—including TP, HF, LF/HF, LF% in AW (open circle and dashed line), and QS (closed circle and solid line) stages—during the light period with the number of platform crosses. Early stage: WT and APP/PS1, n = 17; advanced stage: WT and APP/PS1, n = 10. *p < 0.05 by Pearson correlation analysis. There was no significant correlation between physiological parameters and number of platform crosses by Spearman correlation analysis for nonnormality. AW, active waking; QS, quiet sleep; TP, total power of heart rate variability; HF, high-frequency power of heart rate variability; LF/HF, low-frequency power to high-frequency power ratio of heart rate variability; LF%, normalized low-frequency power of heart rate variability; ln, natural logarithm; nu, normalized units; WT, wild type.

To further clarify the possible relationship between the various physiological indices and cognitive function, linear regression analysis was applied (Figs. 6, S2, and S3). At either the early or the advanced stage of the disease, when all mice (both WT and APP/PS1 mice) were analyzed, there was no correlation between the physiological variables and the discrimination ratio (Fig. S2). In the water maze, increases in the number of platform crosses were correlated with a decrease in interruption (r = -0.566, *p* = 0.018) and an increase of delta power% (r = 0.512, *p* = 0.036) in QS (Fig. 6A), and an increase of LF/HF in AW (r = 0.486, *p* = 0.048) (Fig. 6B) during the light period for early-stage mice. By contrast, at the advanced stage, the increase in the number of platform crosses was correlated with an increase of delta power% in QS (r = 0.779, *p* = 0.008) (Fig. 6A) and an increase of TP and HF in both AW (TP: r = 0.661, *p* = 0.037; HF: r = 0.782, *p* = 0.008) and QS (TP: r = 0.768, *p* = 0.009; HF: r = 0.737, *p* = 0.015) (Fig. 6B) during the light period. We further analyzed the correlation between the number of platform crosses and various physiological indicators in WT and APP/PS1 mice individually. An increase in the number of platform crosses was correlated with an increase in TP (r = 0.654, *p* = 0.029) and LF/HF (r = 0.617, *p* = 0.043) in AW (Fig. S3B) and an increase of delta power% (r = 0.837, *p* = 0.001) in QS (Fig. S3A) during the light period only in early-stage APP/PS1 mice (Fig. S3) and not in advanced-stage APP/PS1 or age-matched WT mice (data not shown), likely due to the small size of those groups.

## Discussion

To the best of our knowledge, only a very few studies have connected sleep, autonomic function, and cognitive function changes in APP/PS1 mice at different stages of the disease. We assessed neuropathological characteristics and cognitive behaviors to confirm the disease stage and simultaneously measured sleep and cardiac autonomic function before the neuropathological and cognitive assessments to explore the roles of these physiological parameters in the pathogenesis and progression of AD. At both the early and advanced stages of the disease, Aβ aggregated in APP/PS1 mice brain and triggered neuroinflammation. Advanced-stage APP/PS1 mice had marked cognitive impairments relative to early-stage mice. Regarding physiological parameters, early-stage APP/PS1 mice had sleep disturbances, including prolonged sleep latency, frequent sleep–wake transition (higher frequency and shorter duration in AW or QS stage), and reduced delta power percentage as well as autonomic dysfunction characterized by high heart rate and low overall autonomic function and parasympathetic function mainly during sleep. Similar findings were observed in the advanced-stage APP/PS1 mice; however, autonomic dysfunction also occurred during wakefulness. Moreover, the interaction between delta power percentage and parasympathetic activity during sleep existed in all mice at both stages of the disease. The results of a correlation analysis of physiological biomarkers and memory performance indicated that memory performance was positively correlated with delta power percentage during QS and sympathetic activity during AW and was negatively correlated with interruptions during QS for early-stage mice; in all advanced-stage mice, memory performance was positively correlated with delta power percentage during QS and with overall autonomic function and parasympathetic function during both the AW and QS stages.

The relationship between sleep and AD is particularly relevant because growing evidence in both human and animal studies indicates that sleep–wake regulation plays a critical role in AD pathogenesis^58,59^. Two-thirds of patients with MCI report subjective sleep disturbance^15^. However, according to a recent systematic review and meta-analysis, changes in partial features of sleep macroarchitecture in MCI are heterogeneous and not well defined, and sleep EEG microarchitecture features were only identified by the small number of studies^13^. The majority of previous studies have revealed that the consistent and evident changes of sleep macroarchitecture in MCI include reduced sleep efficiency and total sleep time, specifically reduced amount of REM sleep, and increased sleep latency and wake after sleep onset^13^. Similar with previous studies, we also found that APP/PS1 mice had sleep onset difficulties at the early stage. However, once they had started to sleep, the sleep structure was similar between the genotypes. This is a typical sleep pattern due to increased anxiety level. Notably, it appears to parallel the first day effect in the Morris water maze task when no pretraining protocol was performed. On the first day of the task, the response is primarily driven by the initial shock of being placed in water, measuring anxiety and panic reactions rather than learning. However, there were no significant differences in sleep architecture during the ZT 0–2 period between WT and APP/PS1 mice at the advanced stage. Instead, we observed significant differences in sleep patterns between early-stage and advanced-stage WT mice, indicating that age may contribute to difficulties in falling asleep or increased susceptibility to anxiety. Moreover, in comparison with control mice, APP/PS1 mice at both the early and advanced stages experienced frequent sleep–wake transitions. Although no difference was observed in QS interruption between groups at the early stage of the disease (consistent with most previous human studies^13^), a negative association between QS interruption and cognitive performance was observed. In contrast to studies reporting reduced REM sleep in patients with MCI^13^ and APP/PS1 mice at 9 months of age^59^, we did not observe differences between APP/PS1 and WT mice both at 4 and 8 months of age. These contradictory findings may be partially explained by different disease stages because some studies have reported that, in the mild stages of the disease, AD patients do not experience a reduction in REM sleep; this reduction emerges at the moderate to severe stages^60,61^. No differences in PS, or REM sleep, were observed in the present study; moreover, the accumulated time of the QS, or NREM stage was not significantly different between APP/PS1 and WT mice at both disease stages. Our findings are consistent with those of the aforementioned meta-analysis suggesting that patients with MCI did not spend less time in the deeper stages of NREM sleep, stage 2, or stage 3 (slow-wave sleep)^13^. These findings are contrary to intuition because slow-wave sleep is essential for memory consolidation^62,63^; thus, a reduction in this sleep might be expected during a neurodegenerative process. However, growing evidence in both human and animal studies indicates that the sleep EEG microarchitecture features, especially delta power (slow-wave activity) during NREM sleep, may provide a more sensitive measure for assessing the functional impact of age-related changes to sleep on cognitive performance^64^. In healthy older adults, the Aβ burden in the medial prefrontal cortex was reported to be significantly correlated with the marked reduction in slow-wave activity during NREM sleep. This reduction was further associated with impaired overnight memory consolidation^65^. Furthermore, patients with MCI spent less time in slow-wave sleep and had less slow-wave activity during sleep compared with controls. This reduction of slow-wave activity during sleep, not the reduction of slow-wave sleep time, was correlated with evening-to-morning changes in declarative memory, suggesting that slow-wave activity plays a critical role in sleep-dependent memory consolidation in patients with MCI^66^. Some animal studies have reported a causal relationship between the sleep–wake cycle and Aβ metabolism before and after the onset of Aβ aggregation in different mouse models of β-amyloidosis, including in APP/PS1 mice^59,67^. The findings indicated that Aβ aggregation disrupts the sleep–wake cycle and diurnal fluctuations of Aβ^59^. Notably, in these animal models of AD, Aβ preferentially aggregates in the prefrontal cortex—the source generator of NREM slow-wave oscillations^67,68^— this is consistent with observations of cognitively normal older adults and AD patients^69,70^. However, data about how changes in slow-wave activity relate to cognitive deficits in animal models of AD pathology are absent. In the present study, we observed that APP/PS1 mice at both the early and advanced stages had a significantly reduced delta power percentage compared with controls. Moreover, this reduction was correlated with worse cognitive function across all mice at both the early and advanced stages; this correlation also occurred in APP/PS1 mice at the early stage alone. These findings jointly imply that Aβ aggregation impairs the generation of slow waves in the prefrontal cortex, which plays a causal role in the progression of cognitive decline in AD.

Many studies have demonstrated that the neurodegenerative process is associated with autonomic function using HRV analysis^39^. However, the relationship between HRV and autonomic dysfunction in AD patients is still controversial; some studies have reported significant differences between AD and control groups^22,71,72^, whereas others have reported nonsignificant results^73,74^. Most studies that have reported significant between-group HRV differences have used HF-HRV analysis, indicating that AD patients have lower parasympathetic activity than healthy people do^39^. Moreover, most previous studies investigating cardiac autonomic function in patients with MCI have reported reduced parasympathetic activity in patients with MCI compared with controls^39^. However, contradictory arguments have been proposed^75^. These studies imply that the contradictory findings are probably due to variations in measurements (duration of physiological recordings and direct vs. indirect assessments), varied study settings (posture), and whether the confounding variables were controlled for^39^. Notably, because the autonomic nervous system is known to have substantial variation across the sleep–wake cycle^40,41^, related studies might yield inconsistent findings depending on whether sleep–wake states were distinguished from each other. However, most related studies have only examined autonomic function during wakefulness with short-term HRV recordings^38^. Only one human study has explored HRV during sleep in patients with MCI and found that parasympathetic regulation during NREM sleep, but not during wakefulness or REM sleep, was impaired in patients with MCI compared with healthy controls^76^. In animal studies, only one study explored the changes of autonomic function during wakefulness with short-term HRV recordings during AD progression and found that APP/PS1 mice had no significant changes in autonomic function at 5 months of age but had significantly higher sympathetic function and lower parasympathetic function after 7 months of age when compared with their age-matched WT mice^77^. However, no relevant findings about autonomic function during sleep have been reported in animal studies. In the present study, we performed long-term, simultaneous, continuous recordings of sleep patterns and cardiac autonomic regulation in APP/PS1 mice at different disease stages for 11 h during the light cycle. We found that APP/PS1 mice at the early stage (4 months of age) before cognitive impairment had increases in heart rate and a reduction of overall autonomic function and parasympathetic function during sleep but not during wakefulness; the same autonomic dysfunction existed not only during sleep but also during wakefulness in APP/PS1 mice with cognitive impairment at the advanced stage (8 months of age). Our findings are consistent with observations in a human study, implying that overall autonomic function and parasympathetic function, especially during sleep, may be an early biomarker for AD detection.

On the other hand, literature to further examine the association between HRV and cognitive function is well established in patients without dementia during wakefulness^38^. Many studies have reported that distinct HRV features (HF, LF, and LF/HF) are associated with cognitive performance in both global cognition and specific cognitive domains; the majority of these studies have revealed associations between parasympathetic dysfunction and worse cognitive performance^38^. However, only 2 human studies have explored the relationship between autonomic function during sleep and cognitive function. They reported that higher parasympathetic activity in slow-wave sleep during a nap was significantly correlated with better sleep-mediated memory performance^78,79^. However, in patients with AD and MCI, the relationship between HRV and cognitive function has not yet been characterized during wakefulness, and whether autonomic function during sleep is related to cognitive performance is unknown. In patients with AD, lower cognitive performance was found to be associated with significantly lower cardiac parasympathetic function, which is highly consistent across studies^71,80,81^, whereas mixed results have been reported for the association between sympathetic function and cognitive performance, as indicated by significant^81^, marginal^80^, or no correlation^71^. Our findings are consistent with these results regarding the association between parasympathetic function during wakefulness and cognitive performance in AD. For all mice at the advanced stage, cognitive performance had a positive correlation with overall autonomic function and parasympathetic function but no significant correlation with sympathetic function during the AW and QS stages. By contrast, one recent study reported that postural changes in sympathetic activity (LF/HF and LF%) were positively correlated with a memory test in MCI^82^. Moreover, by contrast with most studies using short-term recordings of HRV (5 min) in patients without dementia, one study using long-term 24-h ECG recordings to analyze HRV reported that increased sympathetic activity (LF/HF) but not decreased parasympathetic activity (HF) is associated with better cognitive performance^83^. Similarly, in the present study, the long-term HRV analysis results for mice revealed that at the early stage, there was a positive correlation between LF/HF during the AW stage and cognitive performance in all mice (WT and APP/PS1) and in APP/PS1 alone. Moreover, LF% during the AW stage showed a trend of a positive correlation with cognitive performance for all mice at the early stage (r = 0.441, *p* = 0.077) and APP/PS1 at the early stage alone (r = 0.566, *p* = 0.070). These findings imply that autonomic dysfunction, specifically parasympathetic hypoactivation, during sleep precedes the development of cognitive impairment in APP/PS1 mice, and those mice with more hyposympathetic states during wakefulness might have more severe cognitive deficits. In particular, the trend of decreased sympathetic activity (LF/HF and LF%) during wakefulness was observed in APP/PS1 relative to WT mice at the advanced stage. Therefore, future studies should explore changes in sympathetic activity in older APP/PS1 mice with more aggravated cognitive impairments or should directly measure the sympathetic system by using microneurography to understand the complex relationship between the sympathetic and parasympathetic systems in the prodromal phase of AD. This study highlights the importance of the distinction between wake-related and sleep-related autonomic functions, especially in the context of the neurodegenerative process and that parasympathetic dysregulation during sleep may be an early indicator of underlying neurodegenerative pathology.

According to these studies linking sleep, the autonomic nervous system, and cognitive functions, strengthening the interaction between sleep and the autonomic nervous system might benefit sleep-dependent memory consolidation^9^. Nevertheless, the relationship linking the autonomic nervous system with sleep has never been investigated in different age groups or in those with cognitive impairment. We observed that neither accumulated sleep time nor sleep interruption was correlated with cardiac autonomic parameters. By contrast, delta power percentage had a significant positive correlation with parasympathetic activity but not with sympathetic activity during sleep. The relationship between sleep and the autonomic nervous system was consistent across all mice at both the early and advanced stages of the disease. We further detected correlations in WT and APP/PS1 mice separately. The significant positive correlation between delta power percentage and parasympathetic activity was only observed in early-stage WT mice but not in other groups, implying that aging or neurodegeneration might cause the weak interactions between delta power percentage and parasympathetic activity during sleep. Collectively, these findings in the present study can partially explain the conceptual model of a previous study indicating that sleep, autonomic activity, and prefrontal brain function contribute both independently and jointly to memory processing^79^. The proposed model is based on some studies reporting that parasympathetic activation expressed through an increase in vagally-mediated HRV during waking is strongly associated with the neuronal activity of the prefrontal cortex, which in turn regulates cognitive functions^84^. The prefrontal cortex generates slow-wave activity during sleep^70^. Compared with wakefulness, sleep has substantially greater parasympathetic activity^42–44^. Thus, strengthening the interaction between slow-wave activity and parasympathetic activity during sleep in the prefrontal cortex might improve cognitive function and even alter the pathophysiology of AD.

Our study has some limitations. First, based on our previous studies in rats, where we used the telemetry system capable of recording 24-h physiological signals in rats, rats mainly sleep during the light period^85,86^. Therefore, due to the limited battery of the wireless sensor on the mouse, we prioritized the investigation of sleep and autonomic function changes during the light period throughout AD pathogenesis in the present study. However, the effects of physiological parameters during the dark period on cognitive function were not explored. Alzheimer's disease patients have been reported to exhibit disrupted circadian rhythms, experiencing not only insomnia at night but also drowsiness, sleepiness, and agitation during daytime^87^. Therefore, we plan to observe the physiological signals during the dark period in APP/PS1 mice, and further differentiate waking into different levels of activity (such as active waking and quiet waking) to delve deeper into the role of autonomic nervous system during wakefulness in Alzheimer's disease and compare these findings with the experimental results obtained during the light period in the future study. Second, previous literature in human studies has indicated a higher prevalence of AD in females compared to males^88^, and animal studies have also shown more severe symptoms in female AD mice^89^. Some studies have proposed that the gender differences in AD may be related to menopause factor^90^. However, in this study, the role of gender differences in AD progression was not investigated. This is because we plan to examine the pathophysiological mechanisms of gender and menopause factors influencing the progression of AD by dividing female APP/PS1 mice into a sham surgery group and an ovariectomy group with/without estrogen treatment in another study. Third, we only performed histochemistry to confirm Aβ aggregation in the mouse brains at different stages of the disease, but Aβ levels were not precisely measured using an enzyme-linked immunosorbent assay. Therefore, the relationship between physiological parameters and Aβ level could not be determined in this study. However, the correlation analysis between physiological parameters and Aβ levels will be analyzed in another study, which was mentioned in the previous limitation, specifically addressing the impact of gender and menopause factors on the progression of AD. Fourth, the interpretations of different HRV indices in terms of clear differences in autonomic modulation mechanisms, especially LF-related variables, were controversial^36,91,92^. The direct measures of sympathetic outflow or main neurotransmitters of the sympathetic and parasympathetic nervous systems, norepinephrine and acetylcholine, should have been employed; this is a deficiency of the present study. However, using a nonintrusive and nonperturbational method can facilitate evaluating autonomic modulation during sleep without modifying the sleep–wake cycle^93^. Finally, disrupted sleep–wake patterns and autonomic dysfunctions in APP/PS1 mice at the early stage may reflect preclinical neurodegenerative changes in the prefrontal cortex, hypothalamus, and brainstem^26,70,94^; however, these changes were not explored in this study. Further studies are necessary to strengthen our results.

## Conclusions

In this study, we present evidence that sleep disturbances, especially reduced slow-wave activity during sleep, precedes the development of cognitive impairments in APP/PS1 mice. These reductions in slow-wave activity are related to the degree of memory impairment. The same phenomena also exist in the progression to cognitive impairment. Autonomic dysfunction, specifically parasympathetic dysfunction during sleep, was already apparent at the early stages of plaque deposition prior to cognitive impairments. Decreased sympathetic activity during wakefulness might predict worse memory performance. As the disease progresses towards cognitive impairment, (1) parasympathetic function during not only sleep but also wakefulness becomes aberrant and (2) parasympathetic dysfunction during both waking and sleeping might be a powerful predictor of worsening cognitive function. Moreover, understanding the interaction between slow-wave activity and parasympathetic function during sleep might contribute to an evaluation of the severity of disease progression in AD. Overall, we propose a model relating disease stage to AD biomarkers. Aβ biomarkers first become abnormal followed by physiological biomarkers of disrupted slow-wave activity and parasympathetic activity during sleep; these further correlate with cognitive symptom severity. Future studies could extend these findings by quantifying Aβ levels and other molecular neuropathological biomarkers in the brain regions related to sleep–wake cycle regulation and to autonomic and cognitive processes, specifically in the prefrontal cortex; future studies can also examine sleep features and sleep-related HRV in relation to these biomarkers for a more comprehensive and conclusive identification of physiological biomarkers. Our findings elucidate the pathophysiological mechanisms contributing to cognitive decline and the transition to AD; the results aid the development of effective therapeutic interventions in the pathogenesis and progression of AD.

## Supplementary Information

Supplementary Information 1.

## Data Availability

All data generated or analyzed during this study are included in this published article (and its Supplementary Information files).
