# Supplementary material for: Reduced slow-wave activity and autonomic dysfunction during sleep precede cognitive deficits in Alzheimer’s disease transgenic mice
Source: Sci Rep. 2023 Jul 11;13:11231. doi: 10.1038/s41598-023-38214-6 (PMC10336117; doi:10.1038/s41598-023-38214-6)
Supplement: Supplementary file 1 — Supplementary Information 1. [file 41598_2023_38214_MOESM1_ESM.pdf]

Reduced slow-wave activity and autonomic dysfunction during sleep precede cognitive deficits in Alzheimer's disease transgenic mice

Chieh-Wen Chen[1,2,10,†], Yam-Ting Kwok[5,†], Yu-Ting Cheng[1,2], Yu-Shan Huang[7], Terry B. J. Kuo[1,2,3,6,9], Cheng-Han Wu[1,2], Pei-Jing Du[1,2], Albert C Yang[1,3,4,8,*], Cheryl C. H. Yang[1,2,3,6,*]

[1]Institute of Brain Science, [2]Sleep Research Center, [3]Brain Research Center, [4]Digital Medicine and Smart Healthcare Research Center, National Yang Ming Chiao Tung University, Taipei, Taiwan; [5]Department of Neurology, Far Eastern Memorial Hospital, New Taipei, Taiwan; [6]Department of Education and Research, Taipei City Hospital, Taipei, Taiwan; [7]Department of Anesthesiology, [8]Department of Medical Research, Taipei Veterans General Hospital, Taipei, Taiwan; [9]Center for Mind and Brain Medicine, Tsaotun Psychiatric Center, Ministry of Health and Welfare, Nantou, Taiwan; [10]Department of Health and Leisure Management, Yuanpei University of Medical Technology, Hsinchu, Taiwan.

[†]Chieh-Wen Chen and Yam-Ting Kwok contributed equally to writing this article.

Running Title: sleep problems and dysautonomia in AD pathology

*Corresponding author:
Cheryl C. H. Yang, PhD, Institute of Brain Science, Brain Research Center, and Sleep Research Center, National Yang Ming Chiao Tung University, No. 155, Sec. 2, Li-Nong St., Taipei 11221, Taiwan. Tel: +886-2-28267058, Fax: +886-2-28273123. Email: cchyang@ym.edu.tw
Albert C Yang, MD, PhD, Institute of Brain Science, and Digital Medicine and Smart Healthcare Research Center, National Yang Ming Chiao Tung University, No. 155, Sec. 2, Li-Nong St., Taipei 11221, Taiwan. Tel: +886-2-28267995, Fax: +886-2-28273123. Email: accyang@gmail.com

Supplementary Figures

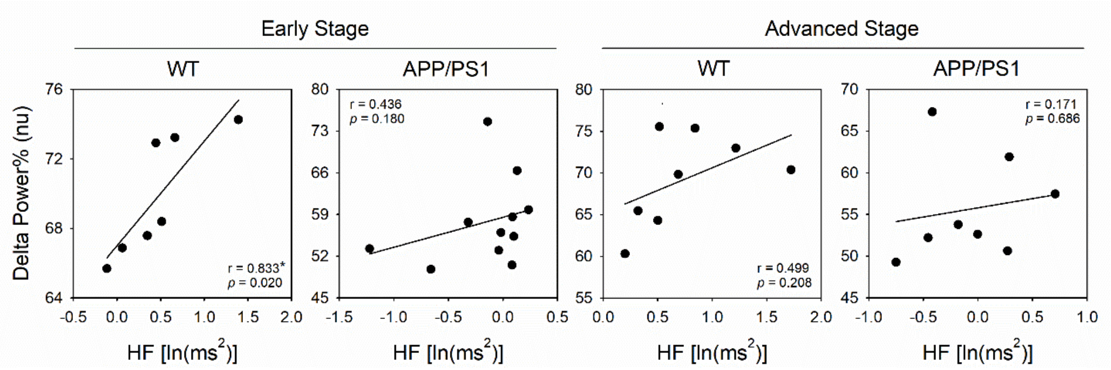

Figure S1. Relationship between HF and delta power percentage of the electroencephalographic spectrogram in quiet sleep during the light period in APP/PS1 mice individually at the early and advanced stages of the disease and their age-matched WT littermates. Early stage: WT, n=7; APP/PS1, n=11; advanced stage: WT, n=8, APP/PS1, n=8. *p<0.05 by Pearson correlation analysis. There was no significant correlation between HF and delta power percentage by Spearman correlation analysis for nonnormality. HF, high-frequency power of heart rate variability; ln, natural logarithm; nu, normalized units; WT, wild type.

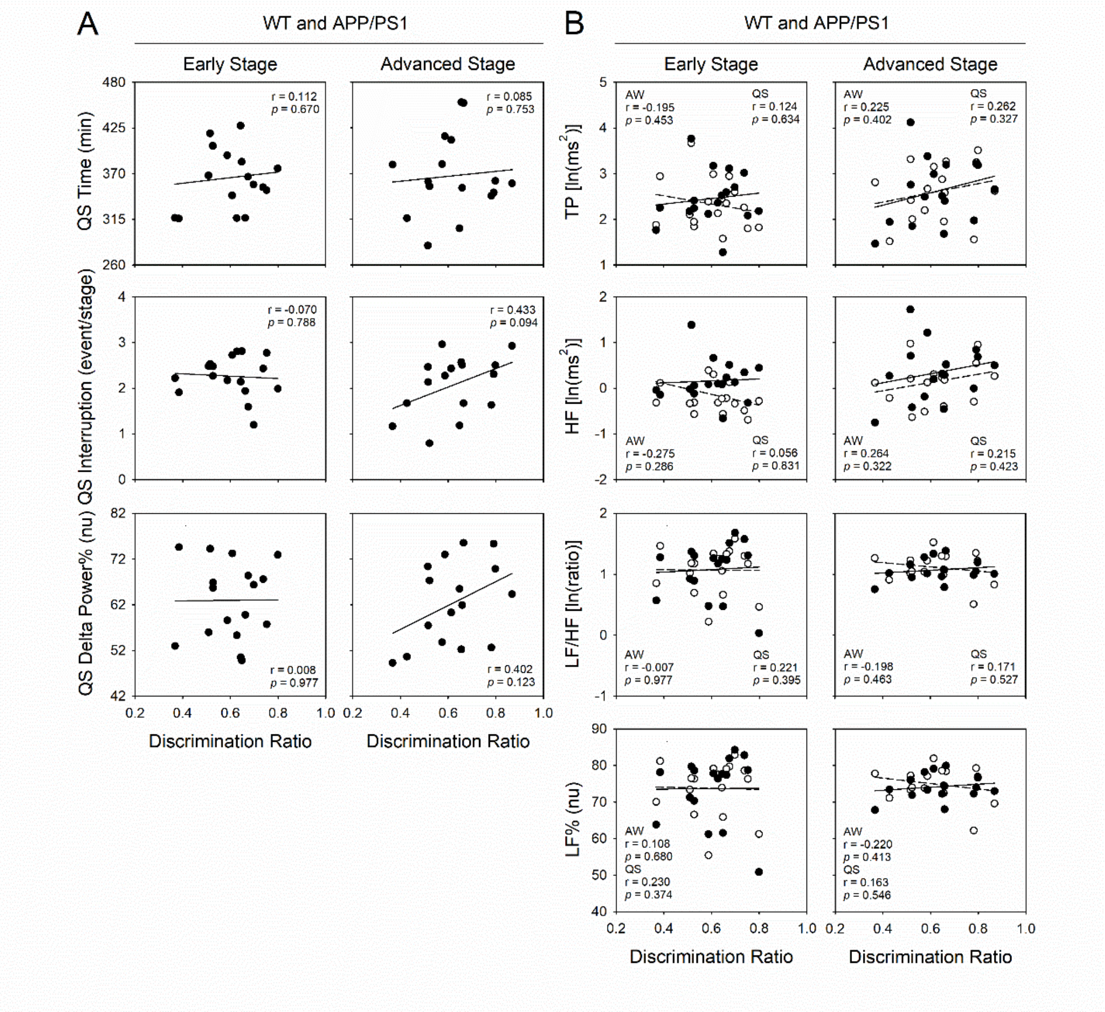

Figure S2. Relationship between physiological parameters and cognitive function in all mice (WT and APP/PS1 mice) individually at the early and advanced stages of the disease. (A) Correlations of accumulated time, interruption and delta power% of the electroencephalographic spectrogram in QS during the light period with the discrimination ratio. (B) The correlation of cardiac autonomic function—including TP, HF, LF/HF, LF% in AW (open circle and dashed line), and QS (closed circle and solid line) stages—during the light period with the discrimination ratio. Early stage: WT and APP/PS1, n=17; advanced stage: WT and APP/PS1, n=16. There was no significant correlation between physiological parameters and discrimination ratio by Pearson correlation analysis for normality and Spearman correlation analysis for nonnormality. AW, active waking; QS, quiet sleep; TP, total power of heart rate variability; HF, high-frequency power of heart rate variability; LF/HF, low-frequency power to high-frequency power ratio of heart rate variability; LF%, normalized low-frequency power of heart rate variability; ln, natural logarithm; nu, normalized units; WT, wild type.

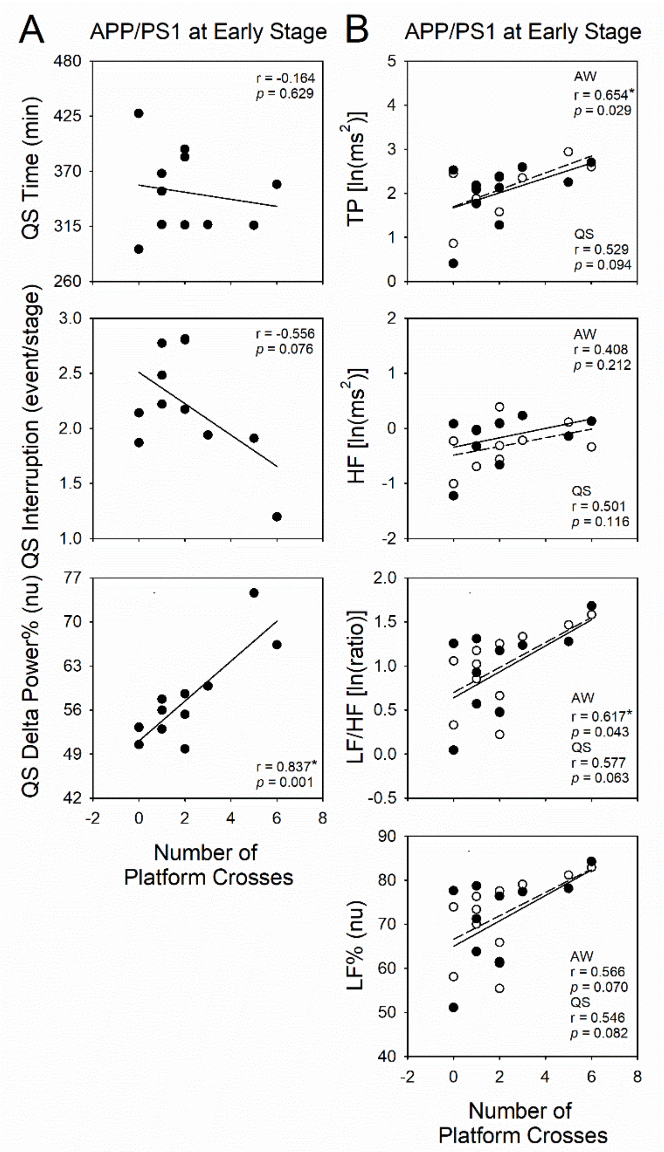

Figure S3. Relationship between physiological parameters and cognitive function in APP/PS1 mice at the early stage of the disease. (A) Correlation of accumulated time, interruption, and delta power% of the electroencephalographic spectrogram in QS during the light period with the number of platform crosses. (B) Correlation of cardiac autonomic function—including TP, HF, LF/HF, LF% in AW (open circle and dashed line), and QS (closed circle and solid line) stages—during the light period with the number of platform crosses. n=11. *p<0.05 by Pearson correlation analysis. There was no significant correlation between physiological parameters and number of platform crosses by Spearman correlation analysis for nonnormality. AW, active waking; QS, quiet sleep; TP, total power of heart rate variability; HF, high-frequency power of heart rate variability; LF/HF, low-frequency power to high-frequency power ratio of heart rate variability; LF%, normalized low-frequency power of heart rate variability; ln, natural logarithm; nu, normalized units; WT, wild type.

Supplementary Tables

Table S1. The number of APP/PS1 mice individually used in each experimental stage, as well as their age-matched WT littermates, and the exclusion criteria.

Experimental Procedure	Early Stage		Advanced Stage		Exclusion Criteria
	WT	APP/PS1	WT	APP/PS1	
Surgery	n=14	n=15	n=14	n=15	
Recovery	n=11	n=13	n=10	n=10	1. Deaths occurred during the surgical procedure.<br>2. Deaths or poor recovery conditions occurred during the recovery period.
Physiological Recording	n=7	n=11	n=8	n=8	1. Poor-quality EEG or EMG signals resulted in difficulties in sleep scoring.<br>2. Poor-quality ECG signals resulted in difficulties in analyzing heart rate variability.<br>3. The connector detached from the mouse's head.<br>4. Seizure was detected and subsequent experiments were terminated.
Novel Object Recognition	n=9	n=10	n=10	n=9	No exploration or low interaction with the objects
Morris Water Maze	n=9	n=12	n=5	n=5	1. Mice do not swim and instead sink or spin in the same place.<br>2. Mice with wounds on their bodies are excluded from participating in this experiment.<br>3. Mice exhibit poor physical condition, weakness, or even death during the course of the experiment.
Histochemistry	n=9	n=10	n=7	n=6	1. The dirty background of histochemistry staining resulted in difficulties in quantification.<br>2. Due to the observed seizures, physical weakness, or deaths during the previous experimental procedures, histochemistry staining was not performed.

EEG, electroencephalogram; EMG, electromyogram; ECG, electrocardiogram; WT, wild type.

Table S2. Thresholds of three variables used for sleep scoring in APP/PS1 mice individually at the early and advanced stages of the disease and their age-matched WT littermates.

Variables	Early Stage		Advanced Stage	
	WT (n=7)	APP/PS1 (n=11)	WT (n=8)	APP/PS1 (n=8)
Intercept	2.295 ± 0.106	2.186 ± 0.122	2.568 ± 0.186	2.404 ± 0.125
Slope	2.314 ± 0.079	2.222 ± 0.107	2.663 ± 0.141	2.554 ± 0.133
Delta Power	6.917 ± 0.212	6.860 ± 0.161	7.163 ± 0.394	7.306 ± 0.162

Values are presented as mean ± SEM. There were no significant differences observed between different mouse strains or between different disease stages by independent *t* test.
